# My Cousin, My Enemy: quasispecies suppression of drug resistance

**DOI:** 10.1016/j.coviro.2016.09.011

**Published:** 2016-10-17

**Authors:** Karla Kirkegaard, Nicholas J van Buuren, Roberto Mateo

**Affiliations:** 1 Department of Genetics, Stanford University School of Medicine, Stanford, CA, United States; 2 Department of Microbiology and Immunology, Stanford University School of Medicine, Stanford, CA, United States

## Abstract

If a freshly minted genome contains a mutation that confers drug resistance, will it be selected in the presence of the drug? Not necessarily. During viral infections, newly synthesized viral genomes occupy the same cells as parent and other progeny genomes. If the antiviral target is chosen so that the drug-resistant progeny’s growth is dominantly inhibited by the drug-susceptible members of its intracellular family, its outgrowth can be suppressed. Precedent for ‘dominant drug targeting’ as a deliberate approach to suppress the outgrowth of inhibitor-resistant viruses has been established for envelope variants of vesicular stomatitis virus and for capsid variants of poliovirus and dengue virus. Small molecules that stabilize oligomeric assemblages are a promising means to an unfit family to destroy the effectiveness of a newborn drug-resistant relative due to the co-assembly of drug-susceptible and drug-resistant monomers.

## Introduction

Darwinian theory postulates two requirements for evolution to occur. First, populations of individuals must display pre-existing genetic diversity. Then, there must be sufficient selection on survival or reproduction that some members of the population and their progeny become increasingly represented. Viruses often evolve quickly due to the large population sizes, high mutation rates, and rapidly changing environments. Dreaded evolutionary events such as adaptation to new hosts or outgrowths of drug-resistant viruses continue to limit human ability to control viral disease.

### Mutational origins of viral diversity in intracellular RNA viral genomes

**Figure 1:**
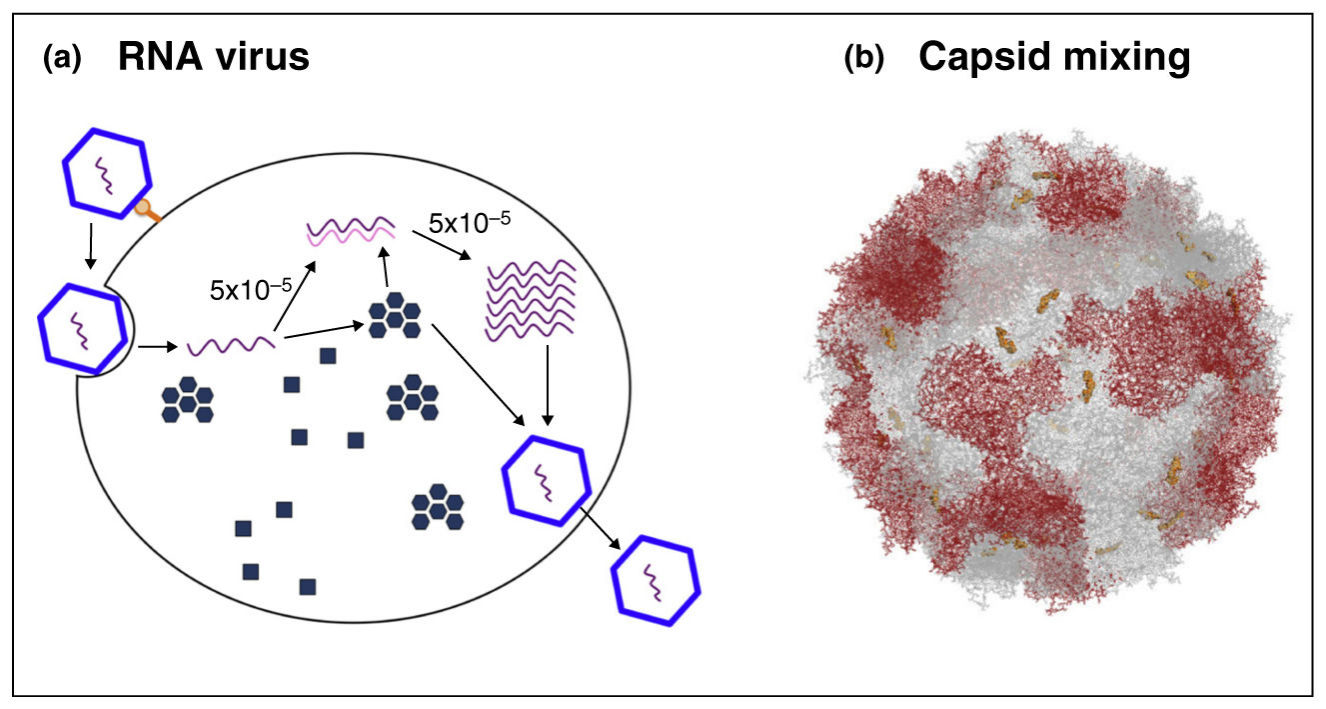
Polyploid genetics of RNA viral infections **(a)** The amplification scheme of a positive-strand RNA virus illustrates the principle of cumulative error frequency, relevant to negative-stranded, double-stranded, and ambisense RNA genomes as well. **(b)** When mutations occur, the intracellular accumulation of all parental and progeny genomes leads to the possibility of mixed oligomer formation, as exemplified by a chimeric poliovirus capsid in which the gray subunits are bound to capsid inhibitor V-073 [10^•^].

The low-fidelity polymerases that copy the genomes of RNA viruses are major contributors to viral diversity. Figure 1a describes the infectious cycle and known mutation frequencies of positive-strand viruses, but the arguments are similar for negative-strand and double-strand RNA viruses. The intrinsic misincorporation frequencies of purified RNA-dependent RNA polymerases during one templating event have been measured to be 3 × 10^−5^ misincorporations/nt for the NS5 polymerase of dengue virus and 5 × 10^−5^ misincorporations/nt for purified 3D polymerases of foot-and-mouth-disease virus and poliovirus [1]. The cumulative mutation frequency after a single infectious cycle within a cell should then be this intrinsic mutation frequency multiplied by the number of templating events [reviewed in [2]].

Once the infecting RNAs are translated, the first intracellular templating event is to copy an original positive strand to a negative strand (Figure 1a). Then, further positive-strand synthesis is templated from the negative strand. In an extreme case, a ‘stamping model’, in which all subsequent positive strands are templated by the original negative strand, the error frequency per cell will be 1 × 10^−4^ mutations/nt, or twice the intrinsic misincorporation frequency. More realistic schemes posit more extended family trees, in which generations of negative and positive strands template each other and the mutation frequency per cell increases linearly with each intracellular generation. Using a clever circulization scheme that minimizes artifactually introduced error, Acevado *et al*. [3^••^] found the cumulative error frequency of poliovirus RNA replication to be 3 × 10^−4^ mutations/nt. This predicts an average of three cycles of negative-strand and positive-strand synthesis during one intracellular infectious cycle. Note that an *intracellular quasispecies* is generated by the infectious cycles of RNA viruses, creating the possibility that generations of RNA genomes that vary slightly in sequence are in close contact. It is from this intracellular viral diversity that newly synthesized drug-resistant genomes are initially selected.

## Approaches to suppressing drug resistance

The diversity of RNA viruses results from these high mutation frequencies and other genetic events such as deletion, recombination, reassortment, transduction of cellular sequences, and the action of cellular editing enzymers. There are two general strategies to thwart the evolution of drug-resistant viruses: by *reducing the frequency* of drug-resistant viruses or *reducing the selection* for their outgrowth.

### Reducing the frequency of drug-resistant viruses

Arguably the most successful strategy for the treatment of highly mutable viruses is *combination therapy*: simultaneous treatment with multiple antivirals. The frequency of variant viruses that are resistant to two or three drugs is the multiple of the frequencies of each drug-resistant variant to each individual drug. HIV-infected and HCV-infected individuals are currently treated with combinations of two to four medications that inhibit viral protein functions or stimulate host responses. These combination therapies are the result of decades of research by hundreds of laboratories.

A second approach to reducing the frequency of drug-resistant viruses is targeting *host molecules* required for viral infections. If an antiviral targets a human protein, for example, it is unlikely that genetic adaptation of host cells to facilitate viral replication would be selected. Sometimes, however, viruses escape drug inhibition by losing their requirement for the targeted host function. For example, HCV growth is dependent on cyclophilins, human proteins whose proline isomerase activities are inhibited by cyclosporin and related compounds. Nonetheless, drug-resistant viral mutations can be selected [4–6]. However, there are many host functions subverted and exploited by viruses and targeting them is a promising approach.

### Reducing the selection of drug-resistant viruses

If an antiviral compound is targeted to a crucial point in an enzyme or complex, it has been argued that drug-resistant viruses will sometimes sustain a high *fitness cost*, becoming so enfeebled by the mutation at these critical residues that they are not selected even in the presence of drug. For example, very low fitness was observed for the few HCV variants isolated from patients who were treated with polymerase inhibitor Sofosbuvir [7]. Nonetheless, high-fitness viruses selected merely for growth were found to be less susceptible to inhibition by Sofosbuvir [8^•^]. Thus, the phenomenon of fitness cost is difficult to predict.

**Figure 2:**
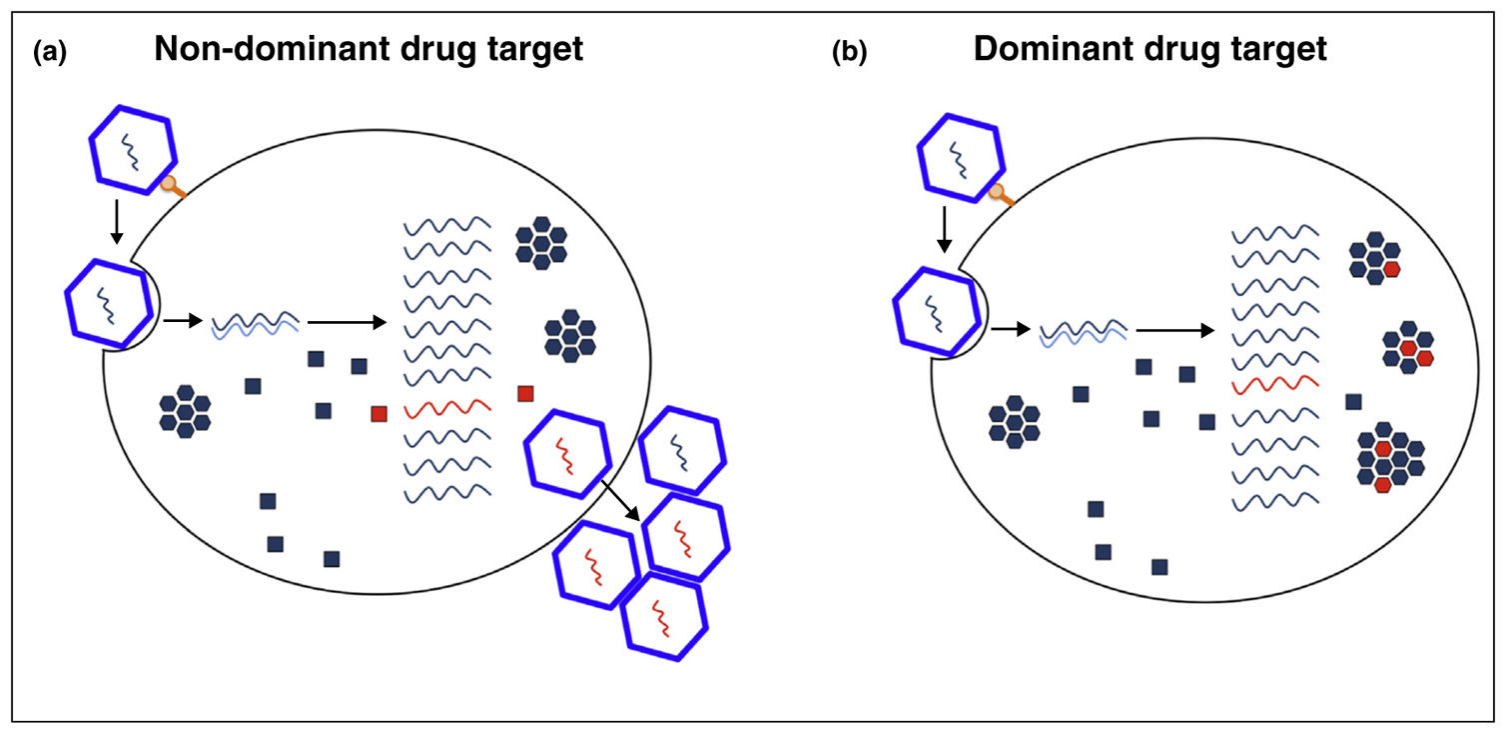
The selection pressures on drug-resistant viruses differ with target biochemistry **(a)** When antiviral targets are trans-acting monomeric proteins or cis-acting molecules [31], production of drug-resistant entities directly benefits replication of the drug-resistant genome. Thus, selection for drug resistance is unimpeded. **(b)** When the drug target is a trans-assembling oligomer, the formation of chimeric oligomers can lead to suppression of the drug-resistant phenotype.

An alternative approach, *dominant drug targeting*, seeks to identify drug targets for which the inevitable drug-resistant mutations arise but are not selected from the intracellular quasispecies. Normally, for a drug target such as a monomeric viral enzyme (Figure 2a), drug resistance is dominant. Resistant genomes will encode resistant enzymes that allow the infectious cycle to proceed for the genome that encodes them. In some cases, resistant products can also provide helper functions that also allow the escape of drug-susceptible viruses and, it is very likely, provide helper functions that allow the escape of drug-susceptible viruses as well.

Many RNA viral products, however, form trans-assembling oligomers such as capsid and matrix constituents and ribonucleoprotein complexes. For oligomeric drug targets as well as monomeric ones, drug-resistant mutations pre-exist in every viral stock and can be selected in tissue culture through passage at low multiplicities of infection (MOIs). However, when a drug-resistant mutation first occurs in an infected cell, it is not alone, but in the presence of many drug-susceptible genomes (Figure 2b). Then, if the protein subunits assemble in trans, oligomers that contain drug-resistant subunits will also contain drug-susceptible ones. If the ratio of susceptible:resistant subunits is high enough, these chimeric structures will be nonfunctional in the presence of the drug and thus *drug susceptibility will be genetically dominant*.

## Effectiveness of dominant drug targeting

The most intuitive examples of a highly oligomeric, trans-assembling assemblage during viral infections are structural proteins such as cores and capsids. The ability of unfit viruses to suppress the growth of more fit viruses present in the same cell was documented for the major surface glycoprotein (G-protein) of vesicular stomatitis virus by the laboratory of Esteban Domingo [9]. Vesicular stomatitis virus variants resistant to neutralization by monoclonal antibodies could be readily recovered by passage in cultured cells at low MOIs. However, when virus was passaged at MOIs of 5 PFU/cell or higher, the recovery of neutralization-resistant variants were suppressed 400-fold. The authors hypothesized that the mixing of G-proteins within cells (1B) led to the ‘phenotypic masking’ of antibody-resistant mutations and prevented their selection. The concept of phenotypic masking is, in more conventional terms, the genetic dominance of the antibody-sensitive over the antibody-resistant phenotype.

**Figure 3:**
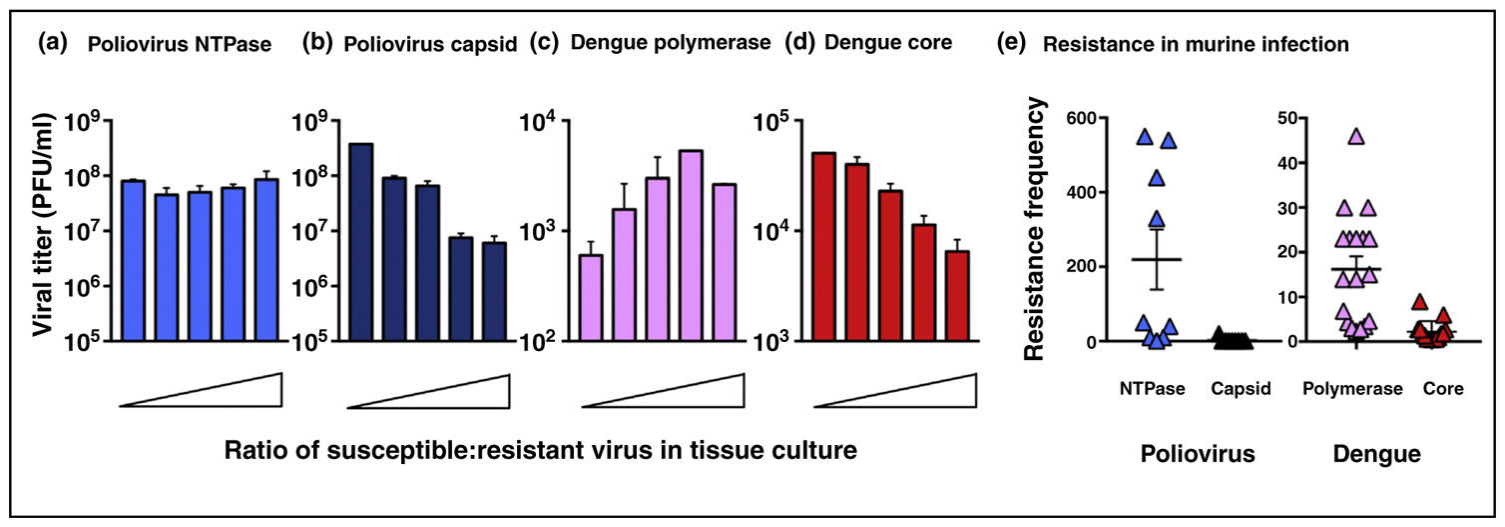
Some antiviral targets allow the selection of drug-resistant variants and some do not **(a)** At low concentrations, guanidine hydrochloride is a potent inhibitor of picornavirus RNA replication, and resistant mutations map to viral NTPase 2C [32]. When cells were infected with a drug-resistant variant in the presence of increasing amounts of drug-susceptible virus, drug resistance is dominant. **(b)** V-073 is a compound being developed for the poliovirus eradication campaign [33] and has 60 binding sites on the poliovirus capsid (Fig. 1b). Drug-susceptibity is dominant. **(c)** MK-0608 is a nucleoside analog that inhibits the active site of hepatitis C, dengue, and zika virus polymerases [34–36]. Drug resistance is dominant. **(d)** ST-148 is a small molecule that hyper-stabilizes dengue core protein oligomers [37,38]. Drug susceptibility is dominant. **(e)** Poliovirus and dengue virus were grown in susceptible mice for five and four days, respectively, in the presence of individual antiviral compounds. Reduced selection for drug resistance was observed for the capsid and core inhibitors.

Like the dominance of antibody-sensitive phenotypes, we reasoned that some drug-susceptible phenotypes might be dominant over newly arising genomes that could encoded potentially drug-resistant phenotypes. To test this hypothesis, the genetic relationships between drug-resistant and drug-susceptible viruses for different drug targets was determined in both poliovirus and dengue virus [10^•^,11^•^,^12^]. For inhibitors that target the active sites of viral enzymes such as poliovirus NTPase (Figure 3a) and the dengue virus RNA-dependent RNA polymerase (Figure 3c), drug-resistance viruses were dominant. However, for inhibitors of the poliovirus capsid (Figure 3b) or dengue virus core proteins (Figure 3d), drug resistance was suppressed when drug-susceptible viruses were present in the same cell. The *dominance of drug susceptibility* in tissue culture correlated with the suppression of drug resistance during growth of these viruses in mouse models (Figure 3e).

To identify other potential dominant drug targets besides capsid proteins, we look to the literature of dominant-negative mutations [13]. A recent contribution to the endogenous retrovirus literature is a description of Refrex-1, a truncated envelope protein encoded in the genomes of domestic cats that confers protection from feline leukemia virus infection by titration of the viral receptor [14]. The genome of thirteen-lined ground squirrels encodes a protein that is highly homologous to bornavirus nucleoprotein; this host restriction factor inhibits exogenous bornavirus infection by incorporating into viral ribonucleoprotein complexes [15^••^]. These examples illustrate two of the mechanisms for molecular dominance by defective proteins described by Herskowitz: competition for a limiting binding partner and inactivation of oligomeric complexes by the formation of chimeras [13].

Competition for limiting resources is likely to be the mechanism by which defective interfering viral particles inhibit the growth of the competent, wild-type genomes. A strategy to deploy engineered defective interfering particles of HIV to fight the infection is a promising and daring idea [12,16]. On the other hand, defective chimera formation is likely to be the mechanism for many dominant-negative effects of defective viral products. Isolated domains of alphavirus, flavivirus, and HIV structural proteins can inhibit virus membrane fusion, presumably by forming mixed oligomers [17–19], and uncleaved precursors of HIV capsids dominantly inhibit viral maturation by hyperstabilizing the highly oligomeric immature capsid [20]. Expression of nonstructural viral proteins can also dominantly inhibit intracellular processes. Mutant influenza PB1 proteins and defective HCV NS5A proteins have both been shown to dominantly interfere with viral replication in cultured cells [21,22].

We have used an approach of co-transfecting wild-type and non-viable mutant RNAs to identify mutations that dominantly inhibit the growth of wild-type poliovirus. Of the several single polymerase mutations tested, only those near the ‘translocation loop’ [23] were found to have dominant negative effects. From these studies, we conclude that promising dominant drug targets in RNA viruses include capsids, cores, replicase functions in initiation and translocation and intramolecularly cleaving proteases [24]. Other oligomers, such as the matrix proteins of enveloped viruses and components of negative-strand ribonucleoprotein complexes are excellent candidates as well.

## Broadening the concept of dominant-drug targeting

Drug-resistant viruses can be selected within an organism at several stages. The initial inoculum, if sufficiently large, can already include mutant genomes resistant to any single antiviral compound. Assuming no previous drug selection, such variants are expected to present at frequencies no greater than 10^−4^ until subject to selection. When viruses are replicating intracellularly, drug-resistant variants will arise within populations of relatives that can help or hinder their growth and selection. This is the stage at which the dominant drug targeting strategy can exert a powerful effect. Any drug-resistant viruses that escape their cell of origin may then have the opportunity to infect new cells in the absence of other family members, which can readily lead to selection for drug resistance. However, many viruses do not spread dispersively, but locally, to neighboring cells. In these cases, any escaping drug-resistant virus is likely to co-infect these neighboring cells with its susceptible cousins, thus continuing the dominant inhibitory effect.

**Figure 4:**
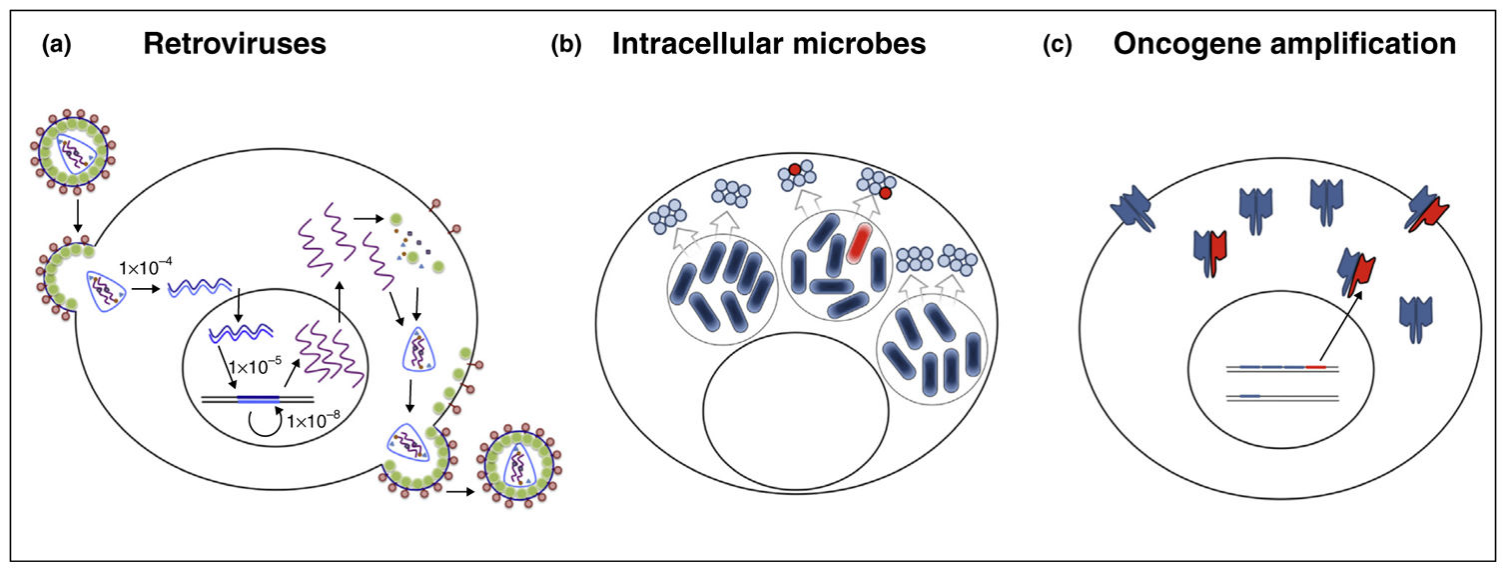
Examples of intercellular and intracellular variation **(a)** Retroviruses synthesize new genomes via cellular transcription, but the most error-prone step is reverse transcription. **(b)** Many bacteria and eukaryotic parasites amplify within cells. Although products within the microbial cells are not shared between organisms, many of the virulence factors that they encode are secreted into a common milieu, creating the possibility of dominant inhibition of drug-resistant variants by drug-susceptible subunits. The oligomeric states of most of these virulence factors are not known. **(c)** The proliferation of cancer cells is often driven by the amplification of oncogenes or growth factors. It is possible that drug resistance could be thwarted should the drug target by an oligomeric product of such amplification events.

How general is the principle of dominant drug targeting? Some other events that give rise to drug resistance arise in polyploid genetic environments. One usually thinks of retroviruses, with their high error frequencies, of being in this category, but retroviral diversity is predominantly extracellular and thus not conducive to the dominant drug targeting strategy. The most error-prone step in the retrovirus infectious cycle is reverse transcription of the two packaged strands of genomic RNA into double-stranded DNA (Figure 4a). The misincorporation frequencies of reverse transcriptases are high: recent measurements of the misincorporation frequency of purified HIV reverse transcriptase by deep sequencing have shown 10^−4^ mis-misincorporations/nt [25]. Subsequent steps — the replication of integrated viral DNA, with the low host misincorporation frequency [(10^−10^/nt) [26]] and the generation of new genomic RNA by host transcription [(10^−5^–10^−6^/nt) [27]] — generate comparatively little intracellular variability. If we assume that retroviral infections of individual cells are mostly initiated by single virions, the genetic diversity will be predominantly *between* infected cells rather than *within* single infected cells.

Intracellular bacteria and eukaryotic parasites that replicate within human cells can, at the error frequency of their DNA polymerases, generate variants that confer resistance to anti-microbial compounds. Such pathogens are known to secrete virulence factors into a shared mileiu (Figure 4b) to hijack host machinery to support their replication and evade innate immune responses. Any of these factors that are oligomeric, such as anthrax toxin [28] could be dominant drug targets.

The effectiveness of most cancer chemotherapies is compromised by drug resistance [reviewed in [29]]. Gene duplication events are common and can correlate with aggressive tumorigenesis and drug resistance. Some oncogenes thus amplified, such as HER2, function as homodimers or heterodimers [30]. As is the case with viruses, drugs that hyperstabilize homo-oligomers have the potential to decrease selection for resistant mutations.
